# Soil salinity impairs soil microbial activity, nutrient availability, plant nutrient uptake, and yield of onion (*Allium cepa* L.)

**DOI:** 10.3389/fpls.2026.1860923

**Published:** 2026-07-15

**Authors:** Mayur B. Patil, Sushant Sukumar Patil, Payal A. Mahadule, Amol R. Pawar, Komal Anil Gade, Thangasamy Arunachalam

**Affiliations:** 1 Department of Agronomy, School of Agriculture, Lovely Professional University, Punjab, Phagwara, India; 2 Department of Soil Science, Indian Council of Agricultural Research (ICAR)-Directorate of Onion and Garlic Research, Pune, Maharashtra, India

**Keywords:** exchangeable sodium, fungal/bacterial ratio, microbial biomass carbon, plant growth, soil enzymatic activity

## Abstract

**Background:**

Onion (*Allium cepa* L.) is highly sensitive to salinity, which can severely affect soil microbial activity, nutrient availability, plant nutrient uptake, and bulb yield.

**Methods:**

A pot experiment was conducted to evaluate the effects of graded salinity levels (0.15–5.00 dS m^-^¹) on four onion cultivars (Bhima Shakti, Bhima Red, Bhima Kiran, and Bhima Swetha) in a factorial completely randomized design with three replications, wherein soil organic carbon, fungal and bacterial population, soil enzyme activity, nutrient availability, plant nutrient uptake and bulb yield were assessed.

**Results:**

Increasing salinity significantly reduced bacterial and fungal populations, fungal-to-bacterial ratio, enzymatic activities, microbial biomass, and nutrient availability. At 5.00 dS m^-^¹, bacterial and fungal populations declined by 17.8% and 44.8%, respectively, while dehydrogenase and alkaline phosphatase activities decreased by 52.3% and 39.1%. Available N, P, S, and B also decreased notably, with P and B showing declines of up to 19.1% and 38.1%. Consequently, plant nutrient uptake declined sharply, with N, P, K, and S decreasing by 34–57%, and micronutrients (Zn, Mn, Cu) by over 80% at 3.55 dS m^-^¹. The cumulative effects of reduced microbial activity and nutrient uptake led to substantial yield losses. Bhima Swetha, Bhima Red, and Bhima Shakti showed 3–10% yield gains at 0.49 dS m^-^¹, whereas onion yield declined sharply above 1.85 dS m^-^¹ and yield losses exceeded 80% at 3.55 dS m^-^¹ across all cultivars.

**Conclusion:**

Bhima Shakti, and Bhima Red increased onion yield slightly at salinity level of 0.49 dS m^-^¹, maintaining higher microbial activity, nutrient uptake, and bulb yield. The study demonstrates that even moderate salinity can substantially impair onion growth and productivity, emphasizing the need for cultivar selection and soil management to mitigate salinity stress.

## Introduction

Onion (*Allium cepa* L.) is one of the most important vegetable crops cultivated worldwide (Thangasamy et al., 2024). India is a leading producer, with an annual production of approximately 30 million tonnes from 1.6 million hectares (FAOSTAT, 2025). Despite this large production area, onion productivity remains relatively low and has declined in recent years due to increasing climatic variability and extreme weather events (van Oort et al., 2023; Jung et al., 2024). Among abiotic stresses, waterlogging during the monsoon season severely affects onion production (Thangasamy et al., 2023), while low water availability and salinity are major constraints during winter cultivation (Alam et al., 2023).

Following the winter season, capillary rise may bring soluble salts leached during the kharif and late kharif seasons back to the soil surface. This process is further intensified by high temperatures and enhanced evaporation, resulting in salt accumulation in the root zone (Liu et al., 2019; Wang et al., 2024). Onion is particularly sensitive to salinity, and even slight increases in soil salinity can adversely affect plant growth and yield (Alam et al., 2023). Increasing soil salinity from 0.68 to 12 dS m^-^¹ has been reported to reduce onion yields by 43–88%, depending on cultivar and duration of exposure (Nassirou et al., 2024). Similarly, García et al. (2015) reported an 18.5% yield loss at 1.4 dS m^-^¹, while Alam et al. (2023) documented yield reductions ranging from 10% to 100% under varying salinity levels. High concentrations of sodium (Na^+^) and chloride (Cl^-^) in the root zone led to ionic toxicity, and poor selectivity of ion transporters between Na^+^ and K^+^ results in excessive Na^+^ accumulation in plant tissues, thereby impairing morpho-physiological, biochemical, and metabolic processes (Rahneshan et al., 2018).

Salinity also modifies soil physico-chemical properties by increasing pH, electrical conductivity, exchangeable sodium percentage (ESP), and sodium adsorption ratio (SAR), while displacing essential nutrients such as K^+^, Ca²^+^, and Mg²^+^ from soil exchange sites (Ghani et al., 2021). Cl^-^ toxicity further disrupts nutrient uptake, leading to deficiencies of nitrogen (N), phosphorus (P), sulfur (S), and boron (B). In addition, salinity negatively affects soil organic carbon (SOC) dynamics, and alters microbial community composition, resulting in reduced microbial biomass, suppressed enzyme activities, impaired organic matter decomposition, and disrupted nutrient cycling processes (Yan et al., 2015; Bai et al., 2020; Ghani et al., 2021; Hou et al., 2021; Boumaaza et al., 2022). These soil-mediated effects compound the negative impact of salinity on crop productivity.

Although previous studies have examined salinity effects on plant growth, physiological responses, and yield in onion and other crops (Sanwal et al., 2022; Alam et al., 2023; Patil et al., 2026), limited information is available on the integrated effects of salinity on soil biological properties, nutrient availability, nutrient uptake, and bulb yield in onion cultivars under graded salinity conditions. Furthermore, the relationship between salinity-induced soil biological changes and crop performance remains poorly understood, particularly for salt-sensitive crops like onion. Addressing these knowledge gaps is essential for developing effective salinity management strategies and identifying tolerant cultivars.

Therefore, the present study aimed to evaluate the effect of graded salinity levels on soil biological properties, nutrient availability, plant nutrient uptake, and bulb yield in onion cultivars. We hypothesized that: (1) increasing salinity would adversely affect soil microbial populations, microbial activity, and nutrient availability; (2) salinity-induced reductions in soil biological activity and nutrient availability would reduce plant nutrient uptake and bulb yield; and (3) onion cultivars would differ in their response to salinity stress. To test these hypotheses, four onion cultivars were evaluated under graded salinity levels, and their effects on soil microbial populations, enzyme activities, nutrient availability, nutrient uptake, and bulb yield were systematically investigated.

## Materials and methods

### Experimental site

A pot experiment was conducted at the farm of ICAR–Directorate of Onion and Garlic Research (ICAR–DOGR), Pune, Maharashtra, India. The site is situated at 18.32° N latitude, 73.51° E longitude, and 645 m above mean sea level. The experiment was conducted during the Rabi season (December 2024–March 2025) under tropical dry–humid climatic conditions. During the study period, mean maximum air temperatures ranged from 27.7 °C to 38.2 °C, while mean minimum temperatures varied between 10.2 °C and 20.2 °C. Prior to the experiment, the soil was analyzed and found to have a clay loam texture with a slightly alkaline pH (7.3–7.9). Moderate levels of SOC (6.7–7.2 g kg^-^¹) and calcium carbonate (4.8–5.3%) were recorded. Available N was low (62.0–72.8 mg kg^-^¹), whereas P (10.8–13.0 mg kg^-^¹) and potassium (K) (230.2–267.0 mg kg^-^¹) were relatively higher (**Supplementary Table 1**).

### Experimental details

The experiment was laid out in a factorial completely randomized design with two factors: six salinity levels and four onion genotypes, resulting in 24 treatment combinations, each replicated three times. The genotypes evaluated were Bhima Shakti, Bhima Red, Bhima Kiran, and Bhima Shweta. Salinity treatments included T1 (control, 0.15 dS m^-^¹), T2 (0.49 dS m^-^¹), T3 (0.85 dS m^-^¹), T4 (1.85 dS m^-^¹), T5 (3.55 dS m^-^¹), and T6 (5.00 dS m^-^¹). Pots of 60 × 40 × 32 cm size, with a holding capacity of 50 kg, were used. Desired salinity levels were created by preparing saline solutions with sodium chloride (NaCl). Before transplanting, full doses of P, K, and S, along with 20% of the N, were applied as basal fertilizers. The remaining 80% of N was top-dressed in three equal splits at 15, 30 and 45 days after transplanting (DAT). Complex fertilizer 10:26:26 (3.46 g pot^-^¹), urea (2.11 g pot^-^¹), muriate of potash (0.74 g pot^-^¹), and elemental S (0.66 g pot^-^¹) were used as fertilizer sources.

Seedlings that were 45 days old were transplanted at a spacing of 15 × 10 cm (row-to-row and plant-to-plant), with twelve plants accommodated in each pot. Irrigation was provided every two days, and weeds were removed manually at 45 DAT. Intercultural operations and plant protection measures were followed as per the ICAR–DOGR package of practices. Soil samples were collected at 15-day intervals to monitor electrical conductivity (EC), and salinity was maintained throughout the crop growth period using NaCl solutions at prescribed concentrations. Plant height and number of leaves were recorded at 30, 60, and 90 DAT. Fully matured leaves (third from the top) were sampled to determine the leaf area index. Harvesting was performed during the first week of April 2025, once 50% neck fall was observed. Bulb size and diameter were measured using a vernier caliper. After three days of curing, bulbs were separated and the average bulb weight was recorded and expressed as g pot^-^¹.

### Soil fungal and bacterial population

Soil bacterial and fungal populations were enumerated using the serial dilution and plate count method (Cappuccino and Sherman, 2014). Serial dilutions (10^-^¹ to 10^-6^) were prepared by suspending 10 g of soil in 95 mL of sterile water, followed by vigorous shaking and sequential transfer of aliquots into sterile dilution blanks. Appropriate dilutions (10^-4^–10^-6^ for bacteria and 10^-3^ for fungi) were plated in sterile Petri dishes. Molten agar medium (~45 °C) was poured, and the plates were allowed to solidify. Incubation was carried out at 28 °C, with plates inverted to prevent condensation (2 days for fungi and 4 days for bacteria). After incubation, colony-forming units (CFU) were enumerated, with spreaders and atypical molds excluded from the counts. CFU were calculated by multiplying number of colonies by the dilution factor, and expressed as CFU g^-^¹ soil.

### Soil enzymatic activities

Soil enzymatic activities were estimated following standard protocols. Dehydrogenase activity was determined by the reduction of triphenyl tetrazolium chloride (TTC) to triphenyl formazan (TPF) as described by Casida et al. (1964). Soil samples were incubated with TTC solution, and the formazan formed was extracted with methanol and quantified spectrophotometrically. Results were expressed as µg TPF g^-^¹ dry soil h^-^¹. Urease activity was assayed following the method of Bremner and Douglas (1971). Soil was incubated with urea solution, and the residual urea after incubation was extracted and determined using a UV-visible spectrophotometer at 527 nm. Enzyme activity was expressed as µg NH_4_–N g^-^¹ soil h^-^¹. Acid and alkaline phosphatase activities were estimated according to Tabatabai and Bremner (1969), using p-nitrophenyl phosphate (pNPP) as substrate in pH-adjusted buffers. The p-nitrophenol (PNP) released was measured spectrophotometrically at 400 nm. Activities were expressed as µg PNP g^-^¹ soil h^-^¹. All enzyme assays were performed under controlled incubation conditions, and values were standardized on a dry soil weight basis.

### Soil microbial biomass carbon and nitrogen

Soil microbial biomass carbon (SMBC) and nitrogen (SMBN) were estimated using the chloroform fumigation–extraction method (Jenkinson and Powlson, 1976; Jenkinson, 1988). Fresh soil samples (10 g) were fumigated with ethanol-free chloroform for 24 h in a vacuum desiccator. Both fumigated and non-fumigated samples were extracted—SMBC with 0.5 M K_2_SO_4_ and SMBN with 2 M KCl. Extractable organic carbon was determined by dichromate oxidation, while ammonium (NH_4_^+^-N) and nitrate (NO_3_^-^-N) were quantified by distillation and titration. SMBC and SMBN were calculated from the difference between fumigated and non-fumigated extracts using standard conversion factors (K_EC = 0.25 for carbon; K_N = 0.50 for N) and expressed as µg C g^-^¹ and µg N g^-^¹ dry soil, respectively.

### Soil sampling and analysis

Soil samples were collected from all pots at harvest and initially before experimentation, from the 0–15 cm depth. Samples were air-dried, sieved (2 mm), and used for physicochemical analyses. Soil pH and EC were measured in a saturated soil paste using a pH meter and EC meter, respectively (Romić et al., 2021). SOC was determined by the Walkley–Black method (Walkley and Black, 1934), and calcium carbonate (CaCO_3_) by titration. Available N, P, K, and S were estimated using the alkaline permanganate, Olsen, 1N ammonium acetate, and 0.15 M CaCl_2_ extraction methods, respectively (Jackson, 1967). Exchangeable calcium (Ca) and magnesium (Mg) were determined by modified EDTA titration, while micronutrients namely iron (Fe), manganese (Mn), zinc (Zn), and copper (Cu) were measured by DTPA extraction and atomic absorption spectrophotometry (Lindsay and Norvell, 1978). B was extracted with hot water and quantified via the azomethine-H method (John et al., 1975). Na^+^ and Cl^-^ were determined using flame photometry and Mohr’s titration, respectively. Cation exchange capacity (CEC) was measured by ammonium acetate extraction (Polemio and Rhoades, 1977), and soil sodicity was assessed using ESP and SAR.

### Plant sampling and analysis

At harvest, five plants were collected from each pot. The samples were thoroughly rinsed with distilled water to remove adhering soil, and bulbs and leaves were separated. Plant parts were chopped, air-dried, and subsequently oven-dried at 60 °C until a constant weight was achieved. Dry matter yield of leaves and bulbs was recorded separately, and combined to calculate plant dry matter. Dried samples were ground, passed through a 2.0 mm sieve, and prepared for nutrient analysis following standard protocols (Jackson, 1967). Total N was determined using the micro-Kjeldahl method. Other nutrients—P, K, S, Ca, Mg, Na, and Cl—were analyzed after di-acid digestion. P was measured by the vanadomolybdate yellow colorimetric method, K and Na by flame photometry, S by the turbidimetric method, and Ca and Mg by EDTA titration. Micronutrients (Fe, Zn, Mn, Cu) were determined using atomic absorption spectrophotometry (Lindsay and Norvell, 1978), while Cl^-^ was analyzed by Mohr’s titration. Total nutrient content was calculated by multiplying nutrient concentration with dry matter per plant.

### Statistical analysis

Soil parameters were analyzed using a single-factor CRD, while yield and nutrient uptake were analyzed using a factorial CRD (treatment × variety). Analysis of variance (ANOVA) was performed in R to test treatment effects, and mean comparisons were made using the Tukey–Kramer HSD test at *P* < 0.05. Pearson correlation was calculated in R to examine pairwise relationships among microbial populations, enzyme activity, soil nutrient availability, nutrient uptake, and yield parameters. Principal component regression (PCR) and partial least squares regression (PLSR) were used to examine relationships between soil physicochemical and biological parameters and onion yield and nutrient uptake under salinity. All variables were standardized (mean = 0, SD = 1) prior to analysis. PCR was performed by applying principal component analysis (PCA) to soil variables, followed by regression of selected components. PLSR was used to directly relate soil variables (X matrix) to crop responses (Y matrix), accommodating multicollinearity. The optimal number of components for both methods was determined using leave-one-out cross-validation based on minimum root mean square error of prediction. Variable importance was assessed using variable importance in projection (VIP) scores, with VIP ≥ 1 considered influential, and the direction of association was inferred from the sign of standardized PLS regression coefficients. All statistical analyses were performed using R using the stats and pls packages (R Core Team, 2025).

## Results

### Fungal and bacterial population

Soil salinity significantly influenced the populations of soil bacteria and fungi, as well as the fungal-to-bacterial ratio (**Table 1**). The control plot exhibited the highest bacterial and fungal populations, along with the highest fungal-to-bacterial ratio, followed by the 0.49 dS m^-^¹ treatment. Increasing the salinity level beyond 0.85 dS m^-^¹ led to a significant decline in both bacterial and fungal populations, as well as in the fungal-to-bacterial ratio. Specifically, at 0.85 dS m^-^¹, bacterial population declined by 5.6% compared to the control. Further increasing the salinity to 1.85, 3.55, and 5.00 dS m^-^¹ reduced bacterial populations by 8.3%, 11.4%, and 17.8%, respectively. Similarly, fungal populations declined by 17.2% at 0.85 dS m^-^¹, 28.6% at 1.85 dS m^-^¹, 36.9% at 3.55 dS m^-^¹, and 44.8% at 5.00 dS m^-^¹ relative to the control. The fungal-to-bacterial ratio followed a similar trend of fungal population dynamics, with reductions of 15.4% at 0.85 dS m^-^¹ and 30.8% at both 3.55 and 5.00 dS m^-^¹ compared to the control.

**Table 1:** Effect of soil salinity on biological and enzymatic activity in soil.

Treatments	Bacteria (×10^–6^ CFU g^-1^)	Fungi (×10^–4^ CFU g^-1^)	F-B ratio	Dehydrogenase (μg TPF g^-^¹ h^-^¹)	Acid-phosphatase (μg PNP g^-^¹ h^-^¹)	Alkaline-phosphatase (μg PNP g^-^¹ h^-^¹)	Urease (μg NH4-N g^-1^ h^-1^)	SMBC (μg g^-1^)	SMBN (μg g^-1^)	QMB
Control	2.22 ± 0.04 a	2.90 ± 0.10 a	0.13 ± 0.00 a	13.7 ± 0.6 a	70.1 ± 1.0 a	200.9 ± 14.2 a	92.7 ± 1.5 a	175.0 ± 3.5 a	72.1 ± 1.8 a	2.4 ± 0.02 ab
0.49 dS m-1	2.16 ± 0.03 a	2.77 ± 0.06 a	0.13 ± 0.00 a	11.0 ± 1.7 a	66.1 ± 0.1.7 a	196.9 ± 6.0 a	79.4 ± 0.6 b	152.8 ± 5.8 ab	65.0 ± 3.6 a	2.3 ± 0.04 b
0.85 dS m-1	2.10 ± 0.02 ab	2.40 ± 0.10 ab	0.11 ± 0.00 ab	9.6 ± 1.0 b	56.7 ± 2.5 b	189.2 ± 9.7 a	76.3 ± 0.0 b	133.5 ± 0.0 b	54.2 ± 0.9 b	2.5 ± 0.01 ab
1.85 dS m-1	2.04 ± 0.02 b	2.07 ± 0.15 b	0.10 ± 0.01 bc	9.2 ± 0.6 b	44.4 ± 1.5 c	136.9 ± 7.2 b	73.1 ± 0.0 bc	106.2 ± 3.5 c	45.3 ± 1.5 bc	2.3 ± 0.00 b
3.55 dS m-1	1.97 ± 0.03 bc	1.83 ± 0.21 b	0.09 ± 0.01 c	7.5 ± 0.6 c	38.7 ± 1.2 cd	115.6 ± 7.0 bc	64.8 ± 0.6 c	98.2 ± 8.7 c	32.8 ± 2.4 cd	3.0 ± 0.05 a
5.00 dS m-1	1.83 ± 0.03 c	1.60 ± 0.30 b	0.09 ± 0.02 c	6.4 ± 0.6 c	30.0 ± 1.5 d	91.6 ± 6.4 c	56.5 ± 0.6 d	73.8 ± 6.4 d	29.7 ± 0.9 d	2.5 ± 0.03 ab
SEM±	0.014	0.06	0.01	0.5	0.9	4.8	0.4	3.3	1.3	0.1
HSD	0.12	0.50	0.03	2.7	6.0	45.3	3.0	18.7	7.1	0.6
p value	<0.0001	<0.0001	0.002	<0.0001	<0.0001	<0.0001	<0.0001	<0.0001	<0.0001	0.02

F-B ratio, Fungal-bacterial ratio; SEM, Standard error mean; HSD, Tukey-Kramer Honestly significant difference; PNP, p-nitrophenol; TPF, Triphenyl formazan; SMBC, Soil microbial biomass carbon; SMBN, Soil microbial biomass nitrogen; QMB, Microbial quotient, Electrical conductivity in control: 0.15 dS m^-1^. Means within a column followed by the same letter are not significantly different according to the Tukey–Kramer honestly significant difference (HSD) test at P ≤ 0.05. The data are presented as mean ± standard deviation (SD) of three replications.

### SMBC and soil enzymatic activity

Salinity levels significantly affected soil enzymatic activities, including dehydrogenase, urease, acid phosphatase, and alkaline phosphatase (**Table 1**). Increasing salinity from 0.49 to 5.00 dS m^-^¹ led to a significant reduction in the activity of all enzymes studied. As salinity increased from 0.85 to 5.00 dS m^-^¹, dehydrogenase activity decreased progressively from 28.9% to 52.3%, while alkaline phosphatase activity declined from 17.7% to 39.1%, relative to the control. Urease and acid phosphatase activities exhibited relatively smaller reductions at lower salinity. At 0.85 dS m^-^¹, urease activity declined by 5.7% and acid phosphatase by 2.0%. However, at higher salinity levels, these reductions became comparatively higher. Urease activity decreased by 19.1% at 1.85 dS m^-^¹, and further to 57.2% at 5.00 dS m^-^¹, compared to the control. Similarly, acid phosphatase activity declined by 5.8% at 0.85 dS m^-^¹, 31.2% at 1.85 dS m^-^¹, 42.5% at 3.55 dS m^-^¹, and 54.4% at 5.00 dS m^-^¹.

Microbial biomass carbon (SMBC) and nitrogen (SMBN) were also significantly reduced with increasing salinity. At 0.49 dS m^-^¹, SMBC and SMBN declined by 12.7% and 9.9%, respectively, relative to the control. SMBC further decreased from 23.7% at 0.85 dS m^-^¹ to 57.8% at 5.00 dS m^-^¹. Similarly, SMBN declined by 24.8% at 0.85 dS m^-^¹ and reached a 58.8% reduction at 5.00 dS m^-^¹ compared to the control. The microbial quotient remained relatively stable across all salinity treatments, from the control up to 5.00 dS m^-^¹.

### Soil physico-chemical properties

Salinity levels significantly influenced soil pH, SOC, and CEC (**Table 2**). Soil pH increased progressively from 7.21 at 0.49 dS m^-^¹ to 8.12 at 5.00 dS m^-^¹. The relative increase in pH ranged from 1.3% at 0.49 dS m^-^¹ to 14.0% at 5.00 dS m^-^¹ compared to the control. In contrast, SOC and CEC declined with increasing salinity. SOC decreased by 10.1% at 0.85 dS m^-^¹ and reached a maximum reduction of 35.4% at 5.00 dS m^-^¹. The decline in CEC was even more pronounced, with a 11.9% reduction at 0.85 dS m^-^¹ and a 61.3% decline at 5.00 dS m^-^¹ compared to the control. Additionally, increasing salinity levels increased concentrations of CaCO_3_, Na^+^, and Cl^-^ in the soil, along with increased ESP and SAR. Notably, at a salinity level of 1.85 dS m^-^¹, both ESP and SAR exceeded critical thresholds that may adversely affect crop growth and soil structure. However, CaCO_3_ content decreased with increasing salinity, showing a reduction of 15.8% at 0.85 dS m^-^¹ and 36.2% at 5.00 dS m^-^¹ compared to the control.

**Table 2:** Effect of soil salinity on soil physico-chemical properties.

Treatment	Soil pH	SOC (g kg^-1^)	CaCO3 (%)	Na+ (cmol(p+) kg^-1^)	Cl- (mmol kg-1)	CEC (cmol (p+) kg^-1^)	ESP (%)	SAR
Control	7.18 ± 0.24 c	7.9 ± 0.02 a	3.67 ± 0.38 b	2.38 ± 0.17 c	1.11 ± 0.04 d	21.87 ± 0.41 a	10.89 ± 0.91 d	1.73 ± 0.12 d
0.49 dS m-1	7.21 ± 0.03 c	7.5 ± 0.01 ab	4.08 ± 0.38 ab	2.61 ± 0.07 c	1.74 ± 0.16 d	19.27 ± 0.44 b	13.55 ± 0.10 d	1.84 ± 0.04 d
0.85 dS m-1	7.34 ± 0.16 bc	7.1 ± 0.01 b	4.25 ± 0.25 ab	3.82 ± 0.46 b	3.16 ± 0.27 c	16.02 ± 0.93 c	23.84 ± 2.46 c	2.86 ± 0.28 c
1.85 dS m-1	7.53 ± 0.37 b	6.8 ± 0.00 bc	4.42 ± 0.29 ab	4.11 ± 0.50 b	4.61 ± 0.13 b	14.53 ± 0.42 d	28.33 ± 3.40 c	3.16 ± 0.36 c
3.55 dS m-1	7.90 ± 0.18 a	6.1 ± 0.01 c	4.75 ± 0.43 a	6.00 ± 0.37 a	6.66 ± 0.33 b	11.11 ± 0.41 e	53.99 ± 2.41 b	4.72 ± 0.28 b
5.00 dS m-1	8.12 ± 0.20 a	5.1 ± 0.01 d	5.00 ± 0.43 a	7.37 ± 0.19 a	9.32 ± 0.51 a	8.47 ± 0.35 f	87.11 ± 3.18 a	6.02 ± 0.20 a
SEM±	0.08	0.04	0.17	0.12	0.16	0.31	1.26	0.08
HSD	0.69	0.4	1.08	1.21	1.42	1.49	10.28	1.01
P value	<0.0001	<0.0001	0.003	0.015	<0.0001	<0.0001	<0.0001	0.0002

EC, Electrical conductivity; SOC, Soil organic carbon; CEC, Cation exchange capacity; SAR, Sodium adsorption ratio; ESP, Exchangeable sodium percentage; HSD, Tukey-Kramer Honestly significant difference. Means within a column followed by the same letter are not significantly different according to the Tukey–Kramer honestly significant difference (HSD) test at P ≤ 0.05. The data are presented as mean ± standard deviation (SD) of three replications.

### Soil available nutrients

Soil salinity significantly influenced the availability of nearly all analyzed soil nutrients, including N, P, K, S, Ca, Mg, B, and Fe, with the exception of Cu, Zn, and Mn (**Table 3**). Increasing salinity markedly reduced the concentrations of available N, P, S, and B compared to the control. Among these, available P showed the highest sensitivity, declining by 9.6% at 0.49 dS m^-^¹ and reaching a 19.1% reduction at 5.00 dS m^-^¹. Similarly, available S decreased by 6.9% at 0.49 dS m^-^¹ and by 23.1% at 3.55 dS m^-^¹. Available B declined by 17.1% at 0.49 dS m^-^¹ and reached a 38.1% reduction at 5.00 dS m^-^¹ compared to the control.

**Table 3:** Effect of salinity on soil available macro- and micronutrients.

Treatment	Available macronutrients						Available micronutrients (mg kg^-1^)				
	(mg kg^-1^)				(cmol(p+) kg^-1^)						
	N	P	K	S	Ca	Mg	B	Cu	Fe	Mn	Zn
Control	74.7 ± 0.4 a	13.6 ± 0.5 a	286.8 ± 11.1 a	34.6 ± 1.3 a	3.10 ± 0.05 ab	0.67 ± 0.00 ab	1.05 ± 0.15 a	2.78 ± 0.08 bc	5.10 ± 0.09a	8.45 ± 1.26 ab	1.05 ± 0.07 ab
0.49 dS m-1	72.8 ± 0.4 a	12.3 ± 0.4 ab	287.8 ± 11.6 a	32.2 ± 0.5 a	3.15 ± 0.05 a	0.86 ± 0.10 a	0.87 ± 0.01 b	2.70 ± 0.06 c	4.69 ± 0.08 ab	6.65 ± 0.56 b	0.91 ± 0.11 c
0.85 dS m-1	72.8 ± 0.3 a	12.1 ± 0.6 ab	273.2 ± 7.5 a	31.1 ± 3.6 ab	3.10 ± 0.05 ab	0.47 ± 0.27 bc	0.84 ± 0.01 b	2.81 ± 0.06 bc	4.60 ± 0.11 ab	8.10 ± 0.56 ab	0.95 ± 0.03 bc
1.85 dS m-1	69.1 ± 0.4 ab	11.9 ± 1.7 ab	261.8 ± 11.2 ab	29.0 ± 1.2 ab	2.97 ± 0.03 bc	0.42 ± 0.08 bc	0.87 ± 0.01 b	2.92 ± 0.08 a	4.40 ± 0.12 bc	8.95 ± 1.61 ab	1.02 ± 0.02 abc
3.55 dS m-1	69.1 ± 0.4 ab	11.7 ± 2.1 b	251.3 ± 7.2 b	26.6 ± 2.2 b	2.90 ± 0.05 c	0.33 ± 0.08 c	0.84 ± 0.00 b	2.91 ± 0.05 ab	4.29 ± 0.10 c	9.31 ± 0.49 a	1.08 ± 0.05 ab
5.00 dS m-1	59.7 ± 0.4 b	11.0 ± 1.8 b	246.0 ± 12.6 b	13.3 ± 1.4 c	2.78 ± 0.03 d	0.22 ± 0.05 c	0.65 ± 0.12 c	2.86 ± 0.06 abc	4.29 ± 0.10 c	9.65 ± 0.53 a	1.10 ± 0.04 a
SEM±	0.8	0.2	2.2	1.2	0.03	0.08	0.04	0.04	0.06	0.55	0.03
HSD	5.6	1.1	35.5	5.6	0.13	0.39	0.21	0.11	0.29	2.65	0.17
P-value	<0.0001	<0.0001	<0.0001	<0.0001	<0.0001	0.002	0.002	0.01	0.007	0.04	0.006

SEM, Standard error mean; HSD, Tukey-Kramer Honestly significant difference. Means within a column followed by the same letter are not significantly different according to the Tukey–Kramer honestly significant difference (HSD) test at P ≤ 0.05. The data are presented as mean ± standard deviation (SD) of three replications.

Available N and K showed relatively smaller reductions at both 0.49 and 0.85 dS m^-^¹. N declined by 2.5% at both 0.49 and 0.85 dS m^-^¹ and by 7.5% at 3.55 dS m^-^¹, while the reduction increased to 20.1% at 5.00 dS m^-^¹. Similarly, K showed a 4.7% reduction at 0.85 dS m^-^¹ and 14.2% at 3.55 dS m^-^¹, with a more substantial decline of 39.1% at 5.00 dS m^-^¹. Interestingly, salinity level of 0.49 dS m^-^¹ slightly increased the availability of K (by 0.3%), Ca (by 0.16%), and Mg (by 28.4%) compared to the control. However, Mg availability sharply declined with increasing salinity beyond 0.49 dS m^-^¹—by 29.9% at 0.85 dS m^-^¹ and 67.2% at 5.00 dS m^-^¹. Like K, Ca concentrations showed only a modest reduction, with a 10.3% decline even at the highest salinity level (5.00 dS m^-^¹). Fe also followed a similar trend, decreasing by 8.0% at 0.49 dS m^-^¹ and 15.9% at 5.00 dS m^-^¹.

In contrast, increasing salinity increased the availability of Cu, Mn, and Zn. Compared to the control, Cu increased by 1.1–5.0%, Mn by 5.9–14.2%, and Zn by 2.9–4.9%. However, the concentrations of all three micronutrients were slightly lower at 0.49 dS m^-^¹ than the control, before increasing at higher salinity levels.

### Nutrient uptake

Salinity levels, genotypes, and their interaction significantly affected the uptake of macro- (**Table 4**) and micronutrients (**Supplementary Table 2**), including Na^+^ and Cl^-^ (**Supplementary Table 3**), in onion. The uptake of N, P, K, and S declined progressively as salinity increased from 0.49 to 5.00 dS m^-^¹. Compared to the control, the uptake of N, P, K, and S decreased by 34.0%–55.3%, 40.0%–57.1%, 25.0%–43.2%, and 37.5%–55.6%, respectively. At 3.55 dS m^-^¹, all genotypes exhibited more than 60% reduction in the uptake of these nutrients, except for K, which showed a reduction ranging from 51.5% in Bhima Shakti to 76.7% in Bhima Kiran. Among the genotypes, Bhima Kiran recorded the highest reduction in NPKS uptake across salinity levels.

**Table 4:** Effect of soil salinity levels on N, P, K, and S uptake of onion genotypes.

Treatments	N (g plant^-1^)				P (g plant^-1^)				K (g plant^-1^)				S (g plant^-1^)			
	Bhima Shweta	Bhima Red	Bhima Shakti	Bhima Kiran	Bhima Shweta	Bhima Red	Bhima Shakti	Bhima Kiran	Bhima Shweta	Bhima Red	Bhima Shakti	Bhima Kiran	Bhima Shweta	Bhima Red	Bhima Shakti	Bhima Kiran
Control	0.47	0.47	0.43	0.36	0.07	0.07	0.07	0.05	0.37	0.32	0.33	0.30	0.09	0.08	0.10	0.09
0.49 dS m-1	0.30	0.37	0.26	0.24	0.05	0.05	0.04	0.04	0.26	0.27	0.22	0.23	0.06	0.06	0.06	0.05
0.85 dS m-1	0.21	0.31	0.23	0.18	0.04	0.04	0.03	0.03	0.21	0.24	0.20	0.19	0.05	0.05	0.05	0.04
1.85 dS m-1	0.15	0.17	0.16	0.06	0.02	0.02	0.02	0.01	0.15	0.15	0.16	0.07	0.03	0.03	0.03	0.01
3.55 dS m-1	0.04	0.02	0.07	0.02	0.01	0.00	0.01	0.00	0.04	0.03	0.09	0.03	0.01	0.00	0.01	0.00
5.00 dS m-1	0.02	0.02	0.02	0.01	0.00	0.00	0.00	0.00	0.03	0.02	0.03	0.02	0.00	0.00	0.00	0.00
Factors	p value		HSD		p value		HSD		p value		HSD		p value		HSD	
Treatment	<0.0001		0.07		<0.0001		0.001		<0.0001		0.05		<0.0001		0.01	
Genotype	<0.0001		0.07		<0.0001		0.001		<0.0001		0.04		<0.0001		0.01	
T×G	0.001		0.08		0.0004		0.001		0.0001		0.05		0.002		0.02	

DAT, Days after transplanting; Electrical conductivity in control: 0.15 dS m^-1^, and HSD: Tukey-Kramer Honestly significant difference.

Similar to macronutrients, the uptake of Ca, Mg, and Fe also declined significantly with increasing salinity, with more than 60% reduction observed across all genotypes. Boron uptake showed a comparatively lower decline in Bhima Red and Bhima Shakti, with less than 35% reduction at 1.85 dS m^-^¹ relative to the control. However, boron uptake declined by more than 50% in all genotypes at 3.55 dS m^-^¹. Bhima Red exhibited relatively lower reductions at mild salinity levels, however at 3.55 dS m^-^¹, the uptake of Zn, Mn, and Cu decreased by more than 80% in all genotypes (**Supplementary Table 4**).

### Bulb weight

**Figure 1:**
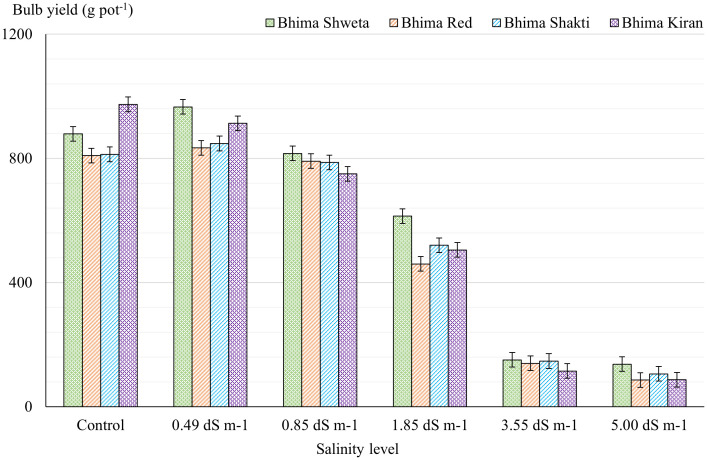
Effect of soil salinity levels on bulb weight of onion genotypes (g pot⁻¹). Values represent means ± standard error of three replications. Factorial ANOVA revealed significant effects of salinity treatment (P < 0.0001), genotype (P < 0.0001), and the treatment × genotype interaction (P = 0.0006) on bulb weight.

Both genotype and salinity levels significantly influenced onion bulb weight (**Figure 1**). Among the tested genotypes, Bhima Swetha, Bhima Red, and Bhima Shakti showed slightly increased bulb yields at a salinity level of 0.49 dS m^-^¹, with yield increase of 9.9%, 3.1%, and 4.3%, respectively, compared to the control. In contrast, Bhima Kiran showed a 6.3% reduction in yield even at 0.49 dS m^-^¹, indicating higher sensitivity to salinity stress. At 0.85 dS m^-^¹, Bhima Red and Bhima Shakti exhibited slight yield reductions of 2.2% and 3.2%, respectively, while Bhima Swetha showed a relatively higher decline of 7.1% compared to the control. As salinity increased to 1.85 dS m^-^¹, all genotypes showed substantial yield losses, ranging from 30.1% in Bhima Swetha to 48.2% in Bhima Kiran. At 3.55 dS m^-^¹, yield reductions exceeded 82% across all genotypes, indicating severe salinity stress effects on bulb development.

### Multivariate relationships among soil, microbial, and plant parameters

**Figure 2:**
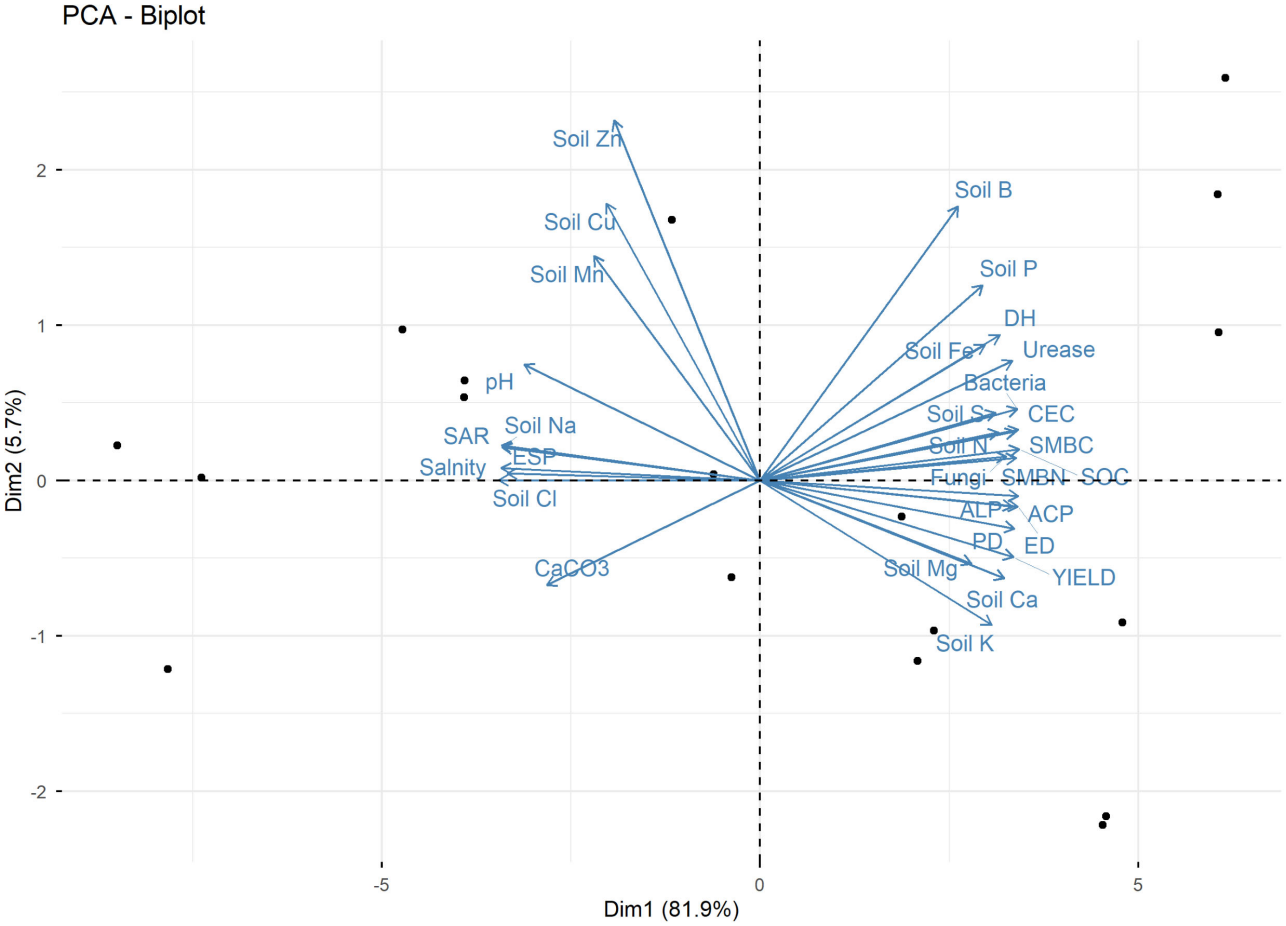
Multivariate relationships of soil microbial and nutrient parameters with onion yield under salinity stress: PCA biplot (n = 18).

**Figure 3:**
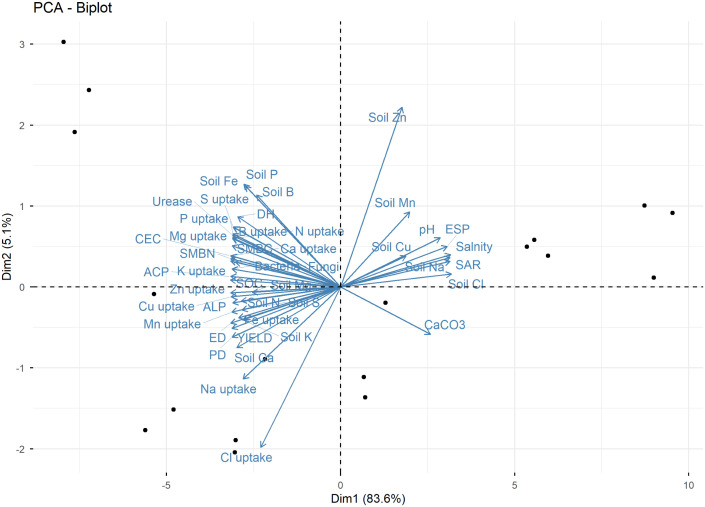
PCA Biplot (PC1 vs PC2) depicting soil, microbial, and nutrient uptake interactions influencing onion yield under salinity stress (n = 18).

Pearson correlation analysis revealed strong positive relationships among soil enzymes (urease, dehydrogenase, phosphatases), SOC, and available macronutrients (N, P, K, S, Fe, B, and Mg), while salinity, soil Na, and Cl showed significant negative correlations with both nutrient availability and plant nutrient uptake (**Supplementary Table 5**). PCA summarized 87.0% of the total variance in the dataset (PC1 = 81.9%, PC2 = 5.1%; **Figure 2**). PC1 showed positive loadings for SOC, enzyme activities, and soil N, P, K, and S, and negative loadings for salinity, soil Na, Cl, Zn, Mn, and Cu, indicating an opposite trend between soil fertility and salinity-induced stress factors. Based on PCA loadings, the most relevant explanatory variables (soil salinity, Na^+^, SOC, dehydrogenase activity, and available N, P, K, S, Fe, and B) and response variables (nutrient uptake of N, P, K, and S, and yield) were selected for PLSR (**Figure 3**). PLSR based on VIP analysis indicated that salinity, soil Na^+^, and soil pH were the most influential factors and showed consistent negative associations with onion yield and N, P, K, and S uptake (**Supplementary Table 6**). SOC and dehydrogenase activity exhibited strong positive associations across all responses. Macronutrients and base cations showed generally positive associations, while their influence was secondary. Micronutrients such as Zn, Mn, B, and Cu displayed low VIP scores and predominantly negative associations.

## Discussion

Onion is highly sensitive to soil salinity, with even low electrical conductivity levels (0.85 dS m^-^¹) adversely affecting growth, bulb development, and quality. This sensitivity distinguishes onion from many other crops that tolerate higher salinity levels. Salinity affects onion through two major pathways. First, higher Na^+^ and Cl^-^ concentrations can disrupt nutrient uptake, assimilation, and metabolic processes. Second, salinity alters the composition and diversity of bacterial and fungal populations in the rhizosphere, thereby affecting nutrient mineralization and availability. While previous studies have mainly focused on plant growth responses to salinity, limited attention has been given to salinity-induced changes in soil microbial communities, nutrient transformations, and their combined influence on nutrient uptake and yield. Therefore, the present study comprehensively evaluated these interrelated effects using four onion cultivars.

### Soil microbial communities and enzyme activity under salinity

Soil bacteria and fungi play a critical role in maintaining soil health through organic matter decomposition, enzyme activity, and nutrient cycling (Bai et al., 2020; Chen et al., 2024). In the present study, both bacterial and fungal populations decreased with increasing salinity. This decline may be associated with reduced biomass production, lower organic matter inputs, and osmotic stress under saline conditions. Similar reductions, ranging from 20–85% for fungi and 40–60% for bacteria, have been reported under high salinity conditions (Li et al., 2021; Boumaaza et al., 2022).

The higher reduction in fungal populations compared with bacterial populations observed in the present study may be related to the higher sensitivity of fungal communities to salinity-induced ionic and osmotic stress, as suggested by previous studies (Liu et al., 2021; Boumaaza et al., 2022). Consequently, the fungal-to-bacterial ratio decreased with increasing salinity, which is consistent with previous findings (Hou et al., 2021; Shakirov et al., 2023). Soil enzyme activities, secreted by both plants and microorganisms, also declined under salinity stress, indicating reduced biological activity and potential limitations in nutrient transformation processes. Additionally, SMBC and SMBN decreased with increasing salinity, suggesting that higher salt levels were associated with lower microbial biomass and activity (Yan et al., 2015).

Furthermore, fungal and bacterial populations, along with microbial activity, showed a significant positive correlation with SOC and a significant negative correlation with soil EC. These associations are consistent with previous reports indicating that saline conditions may adversely affect organic matter turnover and microbial functioning through changes in the soil environment (Sritongon et al., 2022).

### Soil nutrient availability and chemical properties under salinity

Declines in microbial activity and enzyme function under saline conditions may influence nutrient transformations and soil fertility (Pan et al., 2024). Principal component analysis further supported these interactions, as salinity, ESP, and SAR showed negative associations with microbial biomass, enzyme activities, and yield. These results suggest that salinity may be an important factor associated with reduced nutrient mineralization and availability, particularly of N, P, K, S, Fe, and B. High salinity was associated with lower soil available N, which may be related to reduced urease activity and slower mineralization and nitrification processes, as reported in previous studies (Tao et al., 2024). Lower P availability under saline conditions may be associated with reduced mineralization, increased fixation under alkaline conditions, and decreased phosphatase activity, as reported in previous studies (Xie et al., 2022). Similarly, the observed decline in S availability may be related to processes such as competition between Cl^-^ and sulfate ions for adsorption sites, precipitation with Ca²^+^, Mg²^+^, and Fe²^+^ or increased leaching losses under saline conditions (Wang et al., 2019; Zhou et al., 2024).

Lower boron availability observed under saline conditions may be related to reduced release from organic matter under suppressed microbial activity and increased adsorption under saline conditions, as suggested in previous studies (Goldberg et al., 2008; Shireen et al., 2018). The increased ESP and SAR observed in this study are indicative of changes in soil cation balance commonly associated with saline conditions (Gandikota et al., 2025). Similarly, the lower concentrations of K^+^, Ca²^+^, and Mg²^+^ at higher salinity levels may be associated with competitive interactions with Na^+^ and subsequent leaching (Warrence et al., 2003). The observed reduction in Fe availability under higher salinity may be related to mechanisms reported in the literature, including hydroxide precipitation and reduced biological Fe reduction, which can decrease Fe solubility (Tao et al., 2023). In Cl^-^-dominated saline soils, additional ionic interactions may also contribute to reduced Fe uptake. In contrast, Ca, Cu, Zn, and Mn availability remained relatively unaffected, which is consistent with findings reported by Bhaduri et al. (2022) and Hanif et al. (2024).

### Plant nutrient uptake and onion yield

Electrical conductivity showed a significant negative correlation with soil nutrient availability and plant nutrient uptake, whereas soil available nutrients were positively correlated with nutrient uptake. Previous studies have also shown that increasing salinity can impair root structure, causing damage to the epidermis, cortex, and root cap, loss of root hairs, and restricted elongation (Atta et al., 2023). Such changes may reduce the effective root surface area available for nutrient absorption. Salinity-induced osmotic stress has also been reported to limit water and nutrient transport, even at moderate salinity levels (Lu and Fricke, 2023), while Na^+^ may competitively interfere with the uptake of essential cations (Raddatz et al., 2020; Atta et al., 2023). Therefore, the lower nutrient uptake observed under saline conditions in the present study may be associated with a combination of osmotic effects, ionic interactions, and alterations in root function. Reduced uptake of N, P, and K may subsequently contribute to limitations in ATP production and photosynthetic efficiency, potentially resulting in lower growth and biomass accumulation (Volkov, 2015; El and Aboukota, 2019).

Partial least squares regression analysis further indicated that salinity, soil Na^+^, CaCO_3_ content, and soil pH were strongly negatively associated with onion yield and nutrient uptake. These variables were also associated with lower SOC, microbial activity, and nutrient availability, which may be linked to reduced onion productivity. In contrast, SOC showed a strong positive association with yield and nutrient uptake, and was positively associated with microbial activity, nutrient mineralization, and nutrient availability, particularly under low to moderate salinity conditions. Onion yield declined sharply above 1.85 dS m^-^¹, coinciding with reductions in nutrient uptake and changes in soil biological and chemical properties. However, Bhima Shakti and Bhima Red exhibited comparatively higher productivity up to 0.85 dS m^-^¹. This improved performance may reflect differences in physiological and biochemical responses to salinity that were not directly evaluated in the present study (Liu et al., 2015). In contrast, Bhima Kiran showed higher sensitivity, with yield losses occurring even at low salinity levels, possibly due to poor ion homeostasis (Volkov and Beilby, 2017). At higher salinity levels (> 3.55 dS m^-^¹), all genotypes exhibited severe reductions in yield, with yield losses exceeding 85%, consistent with previous reports suggesting that oxidative stress, enzyme inhibition, and impaired assimilate transport may have contributed to lower nutrient uptake and bulb yield under saline conditions. The observed threshold sensitivity was consistent with the findings of Bernstein and Ayers (1953), who reported a decline in onion yield at 1.2 dS m^-^¹ and a 50% yield reduction at 3.9 dS m^-^¹. Similarly, Patil et al. (2026) reported an onion yield reduction of more than 85% at 3.55 dS m^-^¹.

Importantly, this study provides an integrated assessment of soil microbial activity, nutrient availability, and plant responses under graded salinity levels in onion, and examines the associations among salinity-induced changes in soil microbial activity, nutrient availability, nutrient uptake, and bulb yield. Furthermore, the identification of salinity thresholds and differential cultivar responses may help in selecting salt-tolerant onion cultivars and developing effective salinity management strategies for sustainable onion production.

## Conclusion

The present study showed clear associations between soil salinity and changes in soil microbial activity, nutrient availability, nutrient uptake, and onion productivity, as revealed by both PCA and PLSR analyses. Principal component analysis identified soil salinity, Na^+^, and soil pH as variables associated with lower soil microbial biomass, enzyme activity, nutrient availability, and yield. Similarly, PLSR coupled with VIP analysis identified salinity, soil Na^+^, CaCO_3_ content, and soil pH among the most important variables associated with variation in onion yield and N, P, K, and S uptake. In contrast, SOC exhibited positive associations with microbial activity, nutrient mineralization, nutrient availability, and crop performance, particularly under normal conditions. The convergence of PCA and PLSR results suggests that reduced nutrient uptake under saline conditions may be linked to salinity-induced constraints on plant growth and soil biological processes rather than solely to nutrient availability. The observed associations between salinity, reduced microbial activity, altered soil chemical properties, and lower nutrient uptake indicate that multiple interacting factors may contribute to yield reduction under saline conditions. Genotypic differences further showed that cultivars such as Bhima Shakti and Bhima Red maintained comparatively higher productivity under moderate salinity levels, whereas higher salinity was associated with substantial yield losses irrespective of genotype.

Importantly, this study provides an integrated assessment of soil microbial activity, nutrient dynamics, and plant responses under graded salinity levels in onion. Unlike previous studies that primarily focused on plant responses to salinity, the present study examined the associations among salinity-induced changes in soil biological properties, nutrient availability, nutrient uptake, and bulb yield. Furthermore, the identification of salinity thresholds and differential cultivar responses provides valuable insights for the selection of salt-tolerant onion cultivars and the development of effective salinity management strategies for sustainable onion production. These findings can serve as a foundation for future research on microbial interventions and integrated soil management approaches to enhance onion productivity under saline conditions.

## Data Availability

The original contributions presented in the study are included in the article/**Supplementary Material**. Further inquiries can be directed to the corresponding author.
