# Supplementary material for: Soil salinity impairs soil microbial activity, nutrient availability, plant nutrient uptake, and yield of onion (Allium cepa L.)
Source: Front Plant Sci. 2026 Jul 15;17:1860923. doi: 10.3389/fpls.2026.1860923 (PMC13414182; doi:10.3389/fpls.2026.1860923)
Supplement: Supplementary file 1 [file Table1.docx]

Table S1. Initial soil properties recorded before planting

| Soil properties | Control | 0.49 dS m^-1^ | 0.85 dS m^-1^ | 1.85 dS m^-1^ | 3.55 dS m^-1^ | 5.00 dS m^-1^ |
| --- | --- | --- | --- | --- | --- | --- |
| Soil pH | 7.31 | 7.39 | 7.62 | 7.83 | 7.80 | 7.91 |
| EC (dS m^-1^) | 0.11 | 0.34 | 0.72 | 1.61 | 2.93 | 4.10 |
| SOC (%) | 0.67 | 0.66 | 0.68 | 0.66 | 0.70 | 0.72 |
| Calcium Carbonate (%) | 5.00 | 5.17 | 4.88 | 4.83 | 5.33 | 5.17 |
| Nitrogen (mg kg^-1^) | 72.8 | 70.9 | 67.2 | 67.2 | 61.6 | 63.5 |
| Phosphorous (mg kg^-1^) | 13.0 | 12.6 | 11.6 | 11.3 | 10.8 | 10.8 |
| Potassium (mg kg^-1^) | 241.2 | 237.0 | 249.3 | 258.3 | 230.2 | 267.0 |
| Sulphur (mg kg^-1^) | 12.3 | 16.3 | 11.7 | 11.5 | 8.9 | 8.2 |
| Calcium (cmol kg^-1^) | 2.53 | 2.50 | 2.37 | 2.23 | 2.17 | 2.13 |
| Magnesium (cmol kg^-1^) | 0.57 | 0.54 | 0.50 | 0.44 | 0.42 | 0.42 |
| Boron (mg kg^-1^) | 0.80 | 0.71 | 0.63 | 0.63 | 0.64 | 0.66 |
| Copper (mg kg^-1^) | 2.83 | 2.93 | 3.05 | 2.84 | 3.08 | 2.88 |
| Iron (mg kg^-1^) | 4.99 | 4.58 | 4.73 | 4.33 | 4.61 | 4.54 |
| Manganese (mg kg^-1^) | 8.83 | 8.33 | 8.77 | 8.57 | 9.25 | 8.17 |
| Zinc (mg kg^-1^) | 0.85 | 0.83 | 0.88 | 0.85 | 0.94 | 0.91 |
| Sodium (cmol kg^-1^) | 2.58 | 2.81 | 4.40 | 5.09 | 6.97 | 8.35 |
| Chloride (mmol kg^-1^) | 1.18 | 1.16 | 2.06 | 2.03 | 2.95 | 3.20 |
| CEC (cmol(p+) kg^-1^) | 22.42 | 21.98 | 19.70 | 17.10 | 16.28 | 13.63 |
| ESP (%) | 11.49 | 12.78 | 22.36 | 29.74 | 42.89 | 61.36 |
| SAR | 2.07 | 2.28 | 3.68 | 4.40 | 6.13 | 7.38 |

EC: Electrical conductivity, SOC: Soil organic carbon, CEC; Cation exchange capacity, ESP: Exchangeable sodium percentage, SAR: Sodium adsorption ratio
