# Supplementary material for: Soil salinity impairs soil microbial activity, nutrient availability, plant nutrient uptake, and yield of onion (Allium cepa L.)
Source: Front Plant Sci. 2026 Jul 15;17:1860923. doi: 10.3389/fpls.2026.1860923 (PMC13414182; doi:10.3389/fpls.2026.1860923)
Supplement: Supplementary file 2 [file Table2.docx]

Table S2. Effect of soil salinity levels on Ca, Mg, B, and Fe uptake of onion genotypes

| Treatments | Calcium (g plant^-1^) | | | | Magnesium (g plant^-1^) | | | | Boron (mg plant^-1^) | | | | Iron (mg plant^-1^) | | | |
| --- | --- | --- | --- | --- | --- | --- | --- | --- | --- | --- | --- | --- | --- | --- | --- | --- |
|  | Bhima Shweta | Bhima Red | Bhima  Shakti | Bhima Kiran | Bhima Shweta | Bhima Red | Bhima Shakti | Bhima Kiran | Bhima  Shweta | Bhima Red | Bhima Shakti | Bhima Kiran | Bhima Shweta | Bhima Red | Bhima Shakti | Bhima Kiran |
| Control | 0.46 | 0.45 | 0.34 | 0.39 | 0.23 | 0.15 | 0.23 | 0.21 | 9.2 | 5.7 | 5.8 | 7.0 | 79 | 30 | 22 | 45 |
| 0.49 dS m^-1^ | 0.33 | 0.38 | 0.22 | 0.29 | 0.17 | 0.12 | 0.15 | 0.16 | 4.6 | 5.2 | 3.8 | 5.1 | 48 | 18 | 19 | 41 |
| 0.85 dS m^-1^ | 0.25 | 0.31 | 0.18 | 0.21 | 0.13 | 0.11 | 0.14 | 0.12 | 3.5 | 4.4 | 2.8 | 2.2 | 47 | 51 | 20 | 41 |
| 1.85 dS m^-1^ | 0.18 | 0.16 | 0.15 | 0.07 | 0.09 | 0.06 | 0.08 | 0.04 | 2.6 | 2.6 | 1.9 | 0.8 | 51 | 39 | 20 | 15 |
| 3.55 dS m^-1^ | 0.04 | 0.03 | 0.08 | 0.02 | 0.03 | 0.02 | 0.04 | 0.01 | 0.4 | 0.5 | 1.0 | 0.3 | 12 | 8 | 11 | 5 |
| 5.00 dS m^-1^ | 0.02 | 0.02 | 0.02 | 0.02 | 0.02 | 0.01 | 0.02 | 0.01 | 0.2 | 0.3 | 0.3 | 0.2 | 5 | 5 | 3 | 5 |
| Factors | SEM± | | HSD (p=0.05) | | SEM± | | HSD (p=0.05) | | SEM± | | HSD (p=0.05) | | SEM± | | HSD (p=0.05) | |
| Treatment | <0.001 | | 0.07 | | <0.001 | | 0.03 | | <0.001 | | 1.0 | | <0.001 | | 11.9 | |
| Genotype | <0.001 | | 0.06 | | <0.001 | | 0.03 | | <0.001 | | 0.9 | | <0.001 | | 11.1 | |
| T×G | <0.001 | | 0.07 | | <0.001 | | 0.03 | | <0.001 | | 1.0 | | <0.001 | | 12.7 | |

DAT: Days after transplanting, Electrical conductivity in control: 0.15 dS m^-1^, and HSD: Tukey-Kramer Honestly significant difference
