# Supplementary material for: Soil salinity impairs soil microbial activity, nutrient availability, plant nutrient uptake, and yield of onion (Allium cepa L.)
Source: Front Plant Sci. 2026 Jul 15;17:1860923. doi: 10.3389/fpls.2026.1860923 (PMC13414182; doi:10.3389/fpls.2026.1860923)
Supplement: Supplementary file 3 [file Table3.docx]

Table S3. Effect of soil salinity levels on Na^+^ and Cl^-^ uptake of onion genotypes

| Treatment / Genotypes | Sodium | | | | Chloride | | | |
| --- | --- | --- | --- | --- | --- | --- | --- | --- |
|  | Bhima Shweta | Bhima Red | Bhima Shakti | Bhima Kiran | Bhima Shweta | Bhima Red | Bhima Shakti | Bhima Kiran |
| Control | 0.097 | 0.121 | 0.101 | 0.081 | 0.011 | 0.012 | 0.013 | 0.012 |
| 0.49 dS m^-1^ | 0.076 | 0.122 | 0.082 | 0.093 | 0.016 | 0.020 | 0.013 | 0.016 |
| 0.85 dS m^-1^ | 0.094 | 0.174 | 0.079 | 0.112 | 0.020 | 0.024 | 0.017 | 0.019 |
| 1.85 dS m^-1^ | 0.101 | 0.121 | 0.095 | 0.046 | 0.017 | 0.019 | 0.019 | 0.010 |
| 3.55 dS m^-1^ | 0.034 | 0.023 | 0.061 | 0.019 | 0.006 | 0.004 | 0.012 | 0.004 |
| 5.00 dS m^-1^ | 0.024 | 0.021 | 0.021 | 0.018 | 0.004 | 0.004 | 0.005 | 0.004 |
| Factors | p value | | HSD | | P value | | HSD | |
| Treatment | <0.0001 | | 0.03 | | <0.0001 | | 0.005 | |
| Genotype | <0.0001 | | 0.02 | | <0.0001 | | 0.005 | |
| T×G | <0.0001 | | 0.03 | | <0.0001 | | 0.005 | |

DAT: Days after transplanting, Electrical conductivity in control: 0.15 dS m^-1^, and LSD: Least significant difference, SEM±: Standard error mean
