# Supplementary material for: Soil salinity impairs soil microbial activity, nutrient availability, plant nutrient uptake, and yield of onion (Allium cepa L.)
Source: Front Plant Sci. 2026 Jul 15;17:1860923. doi: 10.3389/fpls.2026.1860923 (PMC13414182; doi:10.3389/fpls.2026.1860923)
Supplement: Supplementary file 4 [file Table4.docx]

Table S4. Effect of soil salinity levels on Zn, Mn, and Cu uptake of onion genotypes

| Treatments | Zn (g plant^-1^) | | | | Mn (g plant^-1^) | | | | Cu (g plant^-1^) | | | |
| --- | --- | --- | --- | --- | --- | --- | --- | --- | --- | --- | --- | --- |
|  | Bhima Shweta | Bhima Red | Bhima  Shakti | Bhima Kiran | Bhima Shweta | Bhima Red | Bhima Shakti | Bhima Kiran | Bhima  Shweta | Bhima Red | Bhima Shakti | Bhima Kiran |
| Control | 2.7 | 2.7 | 3.3 | 2.7 | 13 | 6.3 | 10 | 8.6 | 8.0 | 16 | 14 | 13 |
| 0.49 dS m^-1^ | 2.6 | 2.5 | 2.7 | 2.6 | 7.5 | 9.7 | 5.6 | 7.8 | 4.9 | 15 | 8.9 | 11 |
| 0.85 dS m^-1^ | 2.1 | 1.6 | 2.3 | 2.1 | 8.0 | 10 | 6.4 | 7.5 | 5.3 | 14 | 9.1 | 9.8 |
| 1.85 dS m^-1^ | 1.8 | 1.4 | 1.9 | 0.9 | 6.6 | 6.5 | 5.7 | 3.2 | 10 | 8.9 | 7.5 | 3.9 |
| 3.55 dS m^-1^ | 0.5 | 0.3 | 1.2 | 0.3 | 1.9 | 1.0 | 2.8 | 0.8 | 2.7 | 1.6 | 4.3 | 1.3 |
| 5.00 dS m^-1^ | 0.4 | 0.3 | 0.5 | 0.4 | 1.4 | 0.8 | 0.8 | 0.7 | 1.7 | 1.3 | 1.7 | 1.4 |
| Factors | p value | | HSD | | p value | | HSD | | p value | | HSD | |
| Treatment | <0.001 | | 0.7 | | <0.001 | | 2.4 | | <0.001 | | 2.2 | |
| Genotype | <0.001 | | 0.7 | | <0.001 | | 2.2 | | <0.001 | | 2.0 | |
| T×G | 0.17 | | NS | | <0.001 | | 2.6 | | <0.001 | | 2.3 | |

DAT: Days after transplanting, S: Growth stages, Electrical conductivity in control: 0.15 dS m^-1^, and HSD: Tukey-Kramer Honestly significant difference
