# Supplementary material for: Soil salinity impairs soil microbial activity, nutrient availability, plant nutrient uptake, and yield of onion (Allium cepa L.)
Source: Front Plant Sci. 2026 Jul 15;17:1860923. doi: 10.3389/fpls.2026.1860923 (PMC13414182; doi:10.3389/fpls.2026.1860923)
Supplement: Supplementary file 6 [file Table6.docx]

Table S6. Variable importance in projection (VIP) scores identifying the most and less influential soil parameters governing onion yield and nutrient uptake under salinity stress

| Soil parameters | Yield | | | N uptake | | | VIP score | | | Influence for PKS | Direction for PKS |
| --- | --- | --- | --- | --- | --- | --- | --- | --- | --- | --- | --- |
|  | VIP score | Influence | Direction | VIP score | Influence | Direction | P uptake | K uptake | S uptake |  |  |
| Salinity | 1.20 | Most influential | Negative | 1.13 | Most influential | Negative | 1.13 | 1.16 | 1.13 | Most influential | Negative |
| DH | 1.06 | Most influential | Positive | 1.16 | Most influential | Positive | 1.17 | 1.14 | 1.18 | Most influential | Positive |
| pH | 1.08 | Most influential | Negative | 1.02 | Most influential | Negative | 1.02 | 1.04 | 1.01 | Most influential | Negative |
| SOC | 1.16 | Most influential | Positive | 1.15 | Most influential | Positive | 1.15 | 1.16 | 1.15 | Most influential | Positive |
| CaCO3 | 0.94 | Less influential | Negative | 0.95 | Less influential | Negative | 0.98 | 0.96 | 0.99 | Less influential | Negative |
| Soil N | 1.01 | Most influential | Positive | 0.99 | Less influential | Positive | 0.98 | 1.00 | 0.99 | Less influential | Positive |
| Soil P | 0.92 | Less influential | Positive | 1.10 | Most influential | Positive | 1.10 | 1.05 | 1.12 | Most influential | Positive |
| Soil K | 1.06 | Most influential | Positive | 1.05 | Most influential | Positive | 1.05 | 1.05 | 1.04 | Most influential | Positive |
| Soil S | 1.00 | Most influential | Positive | 0.97 | Less influential | Positive | 0.98 | 1.00 | 0.97 | Less influential | Positive |
| Soil Ca | 1.15 | Most influential | Positive | 1.07 | Most influential | Positive | 1.06 | 1.09 | 1.05 | Most influential | Positive |
| Soil Mg | 0.98 | Less influential | Positive | 0.94 | Less influential | Positive | 0.94 | 0.93 | 0.93 | Less influential | Positive |
| Soil B | 0.78 | Less influential | Positive | 0.94 | Less influential | Positive | 0.93 | 0.90 | 0.95 | Less influential | Positive |
| Soil Na | 1.17 | Most influential | Negative | 1.14 | Most influential | Negative | 1.14 | 1.15 | 1.13 | Most influential | Negative |
| Soil Zn | 0.80 | Less influential | Negative | 0.53 | Less influential | Negative | 0.52 | 0.60 | 0.50 | Less influential | Negative |
| Soil Fe | 0.98 | Less influential | Positive | 1.15 | Most influential | Positive | 1.16 | 1.11 | 1.18 | Most influential | Positive |
| Soil Mn | 0.79 | Less influential | Negative | 0.68 | Less influential | Negative | 0.66 | 0.69 | 0.64 | Less influential | Negative |
| Soil Cu | 0.74 | Less influential | Negative | 0.76 | Less influential | Negative | 0.74 | 0.72 | 0.73 | Less influential | Negative |

DH: Dehydrogenase activity. SOC: Soil organic carbon
